# Near-infrared spectroscopy after out-of-hospital cardiac arrest

**DOI:** 10.1186/s13054-019-2428-3

**Published:** 2019-05-14

**Authors:** Pekka Jakkula, Johanna Hästbacka, Matti Reinikainen, Ville Pettilä, Pekka Loisa, Marjaana Tiainen, Erika Wilkman, Stepani Bendel, Thomas Birkelund, Anni Pulkkinen, Minna Bäcklund, Sirkku Heino, Sari Karlsson, Hiski Kopponen, Markus B. Skrifvars

**Affiliations:** 1 Department of Anaesthesiology, Intensive Care and Pain Medicine, University of Helsinki and Helsinki University Hospital, Helsinki, Finland; 2 Department of Anaesthesiology and Intensive Care, University of Eastern Finland and Kuopio University Hospital, Kuopio, Finland; 3 Department of Intensive Care, Päijät-Häme Central Hospital, Lahti, Finland; 4 Department of Neurology, University of Helsinki and Helsinki University Hospital, Helsinki, Finland; 5 Department of Intensive Care, Kuopio University Hospital, Kuopio, Finland; 6 Aarhus University Hospital, Aarhus, Denmark; 7 Department of Intensive Care, Central Finland Central Hospital, Jyväskylä, Finland; 8 Department of Anaesthesiology and Intensive Care, North Karelia Central Hospital, Joensuu, Finland; 9 Department of Intensive Care, Tampere University Hospital, Tampere, Finland; 10 Department of Emergency Medicine and Services, University of Helsinki and Helsinki University Hospital, Helsinki, Finland

**Keywords:** Cardiac arrest, Cerebral oxygenation, Hypoxic ischemic encephalopathy, Intensive care, Neuron-specific enolase (NSE)

## Abstract

**Background:**

Cerebral hypoperfusion may aggravate neurological damage after cardiac arrest. Near-infrared spectroscopy (NIRS) provides information on cerebral oxygenation but its relevance during post-resuscitation care is undefined. We investigated whether cerebral oxygen saturation (rSO_2_) measured with NIRS correlates with the serum concentration of neuron-specific enolase (NSE), a marker of neurological injury, and with clinical outcome in out-of-hospital cardiac arrest (OHCA) patients.

**Methods:**

We performed a post hoc analysis of a randomised clinical trial (COMACARE, NCT02698917) comparing two different levels of carbon dioxide, oxygen and arterial pressure after resuscitation from OHCA with ventricular fibrillation as the initial rhythm. We measured rSO_2_ in 118 OHCA patients with NIRS during the first 36 h of intensive care. We determined the NSE concentrations from serum samples at 48 h after cardiac arrest and assessed neurological outcome with the Cerebral Performance Category (CPC) scale at 6 months. We evaluated the association between rSO_2_ and serum NSE concentrations and the association between rSO_2_ and good (CPC 1–2) and poor (CPC 3–5) neurological outcome.

**Results:**

The median (inter-quartile range (IQR)) NSE concentration at 48 h was 17.5 (13.4–25.0) μg/l in patients with good neurological outcome and 35.2 (22.6–95.8) μg/l in those with poor outcome, *p* < 0.001. We found no significant correlation between median rSO_2_ and NSE at 48 h, *r*_*s*_ = − 0.08, *p* = 0.392. The median (IQR) rSO_2_ during the first 36 h of intensive care was 70.0% (63.5–77.0%) in patients with good outcome and 71.8% (63.3–74.0%) in patients with poor outcome, *p* = 0.943. There was no significant association between rSO_2_ over time and neurological outcome. In a binary logistic regression model, rSO_2_ was not a statistically significant predictor of good neurological outcome (odds ratio 0.99, 95% confidence interval 0.94–1.04, *p* = 0.635).

**Conclusions:**

We found no association between cerebral oxygenation measured with NIRS and NSE concentrations or outcome in patients resuscitated from OHCA.

**Trial registration:**

ClinicalTrials.gov, NCT02698917. Registered on 26 January 2016.

**Electronic supplementary material:**

The online version of this article (10.1186/s13054-019-2428-3) contains supplementary material, which is available to authorized users.

## Background

Cerebral hypoperfusion may lead to diminished cerebral oxygenation after initially successful resuscitation from cardiac arrest (CA). Inadequate oxygenation plausibly aggravates the developing neurological injury [1]. Near-infrared spectroscopy (NIRS) provides a non-invasive technique to assess cerebral oxygenation [2], and the changes in regional cerebral oxygen saturation (rSO_2_) are considered to reflect changes in the relationship between oxygen delivery and consumption in the brain [3]. An increasing body of evidence suggests that optimised cerebral oxygenation is associated with favourable neurologic outcome in a variety of perioperative settings [4]. In addition, higher rSO_2_ is associated with increased probability of return of spontaneous circulation (ROSC) during cardiopulmonary resuscitation (CPR) [5]. However, the clinical relevance of NIRS during post-resuscitation care remains undefined.

In previous studies including both in-hospital and out-of-hospital cardiac arrest (OHCA) patients, higher rSO_2_ values during the early post-resuscitation care were associated with better neurological outcome [6, 7]. On the contrary, in studies including only OHCA patients, the results have been conflicting [8, 9]. We recently published the results of a randomised pilot trial (Carbon dioxide, Oxygen and Mean arterial Pressure After Cardiac Arrest and REsuscitation (COMACARE)) comparing the effect of different levels of arterial carbon dioxide tension (PaCO_2_), arterial oxygen tension (PaO_2_) and mean arterial pressure (MAP) on surrogate markers of brain injury and on cerebral oxygenation after OHCA. We found that both high-normal PaCO_2_ and moderate hyperoxia increased rSO_2_ values but the serum neuron-specific enolase (NSE) concentration was unaffected [10], and the MAP level had no impact on either rSO_2_ or NSE [11].

In the current study, a post hoc analysis of the COMACARE trial, we assessed the associations between cerebral oxygenation and serum neuron-specific enolase (NSE) concentrations and between cerebral oxygenation and neurological outcome in patients resuscitated from ventricular fibrillation (VF) or ventricular tachycardia (VT).

## Methods

All patients included in this post hoc sub-study were participants of the COMACARE trial (NCT02698917). The COMACARE trial protocol and the main results have been published previously [10–12]. In brief, 123 unconscious, mechanically ventilated patients resuscitated from witnessed OHCA with VF or VT as the initial rhythm were randomly assigned to one of eight arms with each arm having a different combination of targets for PaCO_2_, PaO_2_ and MAP. All patients were treated with targeted temperature management (TTM), and the randomisation was stratified according to the target temperature (33 °C or 36 °C). The intervention was continued for 36 h from intensive care unit (ICU) admission or until the patient was extubated or ventilation was set to a spontaneous mode.

The study protocol was approved by the research ethics committees of the Northern Savo Hospital District, Finland (decision No. 295/2015), and the Midtjylland region, Denmark (decision No. 1-10-72-163-16). In addition, the trial protocol was approved by the institutional review board at each site. Because of the nature of the trial, the patients’ unconscious state and the need for a timely intervention, obtaining prior informed consent from the participants at the time of randomisation was not possible. Therefore, a deferred consent procedure was approved by the research ethics committee. We randomised the patients and initiated the intervention at the time of ICU admission and obtained deferred informed consent from the patients’ next of kin afterwards as soon as possible. In addition, we obtained informed consent from all patients who regained sufficient neurological function for independent decision-making [Cerebral Performance Category (CPC) 1–2] after the intervention period.

We measured rSO_2_ with continuous NIRS during the first 36 h of intensive care using a Covidien INVOS 5100C device (Covidien Company, USA). Two non-invasive skin sensors, one to each side, were attached to the patient’s forehead by a study nurse according to the instructions of the manufacturer. As NIRS monitoring was not part of routine care in the participating ICUs, the treating personnel were blinded from the rSO_2_ values. Thus, NIRS monitoring results did not affect patient care. We saved the rSO_2_ values (approximately 10 measurements per minute) from both sensors to a USB memory stick attached to the device. We calculated the hourly medians of these rSO_2_ values of the left channel and used them in the analyses.

The primary endpoint in this study was serum NSE concentration at 48 h after CA. We obtained blood samples for the analyses of the NSE concentrations upon ICU admission and 24 h, 48 h and 72 h after CA. In the Finnish centres, the samples were centrifuged (2000 *g*, 10 min) and the serum was frozen at − 70 °C at the hospital laboratory. The measurements of the NSE concentrations were done in ISLAB laboratories (Kuopio, Finland) using a COBAS e601 line (Hitachi High Technology Co, Tokyo, Japan) with an electrochemiluminescent immunoassay kit (Roche Diagnostics GmbH, Mannheim, Germany) in January 2018. Because of possible interference of haemolysis with NSE results, all serum samples were tested for haemolysis using the Roche haemolysis index [13], and all samples with a haemolysis index ≥ 50 (corresponding to 500 mg of free haemoglobin per litre) (*n* = 7) were excluded from the NSE analyses. At the Aarhus University Hospital, Denmark, the blood samples were analysed immediately by the local laboratory using the same kits as those used by the ISLAB laboratory.

The secondary endpoint was neurological outcome assessed with the CPC scale at 6 months after CA. We defined CPC 1–2 as good outcome and CPC 3–5 as poor outcome. One experienced neurologist, blinded to study group allocation, interviewed all patients or their next of kin by telephone and determined the neurologic outcome using the CPC scale.

### Statistical methods

We compared the categorical data between patients with good and poor outcome by using the Chi-square test. We tested the normality of the continuous data with the Kolmogorov-Smirnov test and compared the data with a normal distribution with the Student *t* test and the data with a non-normal distribution with the Mann–Whitney *U* test (MWU). We compared the median NSE concentrations at 48 h after CA between patients with good and poor neurological outcome at 6 months using the MWU. We calculated the median rSO_2_ during the first 36 h of intensive care for each patient and then assessed the possible relationship between the median rSO_2_ and NSE concentrations at 24, 48 and 72 h using a scatterplot and Spearman’s rank-order correlation. We compared the 36-h medians between patients with good and those with poor outcome using the MWU. We compared the rSO_2_ over time between patients with good and those with poor outcome using a generalised mixed model with a compound-symmetry covariance matrix. We used binary logistic regression model to ascertain the effects of baseline factors and rSO_2_ on the likelihood of good neurological outcome. We chose to model those factors that were significantly associated with prognosis (*p* < 0.05) in univariate analysis. These factors were age, bystander-initiated resuscitation, delay from collapse to return of spontaneous circulation (ROSC) and Acute Physiology and Chronic Health Evaluation (APACHE) II score (Table 1). Finally, we divided the cohort into tertiles according to the lowest 60-min median rSO_2_ during the 36-h period and calculated the probability for a good outcome with 95% confidence interval for each tertile. In addition, we calculated the area under the receiver operating characteristic curve with 95% confidence interval for the lowest 60-min median rSO_2_ to predict good outcome in each tertile. We performed all statistical analyses with SPSS version 24.0 (IBM Corporation, USA).

**Table 1:**
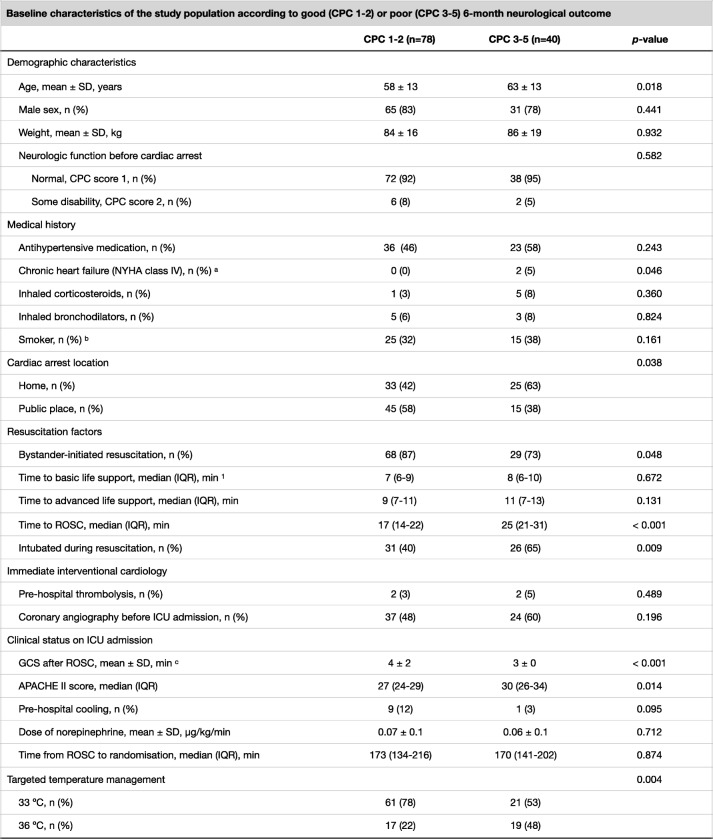
Baseline characteristics of the study population according to good (CPC 1–2) or poor (CPC 3–5) 6-month neurological outcome

*CPC* Cerebral Performance Category [1, good cerebral performance (normal life); 2, moderate cerebral disability (disabled but independent); 3, severe cerebral disability (conscious but disabled and dependent); 4, coma or vegetative state (unconscious); 5, brain death]; *SD* standard deviation, *IQR* inter-quartile range; *NYHA* New York Heart Association; *CPR* cardiopulmonary resuscitation; *ICU* intensive care unit; *GCS* Glasgow Coma Scale; *ROSC* return of spontaneous circulation; *APACHE* Acute Physiology and Chronic Health Evaluation

^a^Data missing for 2 patients

^b^Data missing for 13 patients

^c^Data missing for 9 patients

^1^The time for a paramedic unit with BLS equipment and skills to reach the patient

## Results

**Fig. 1:**
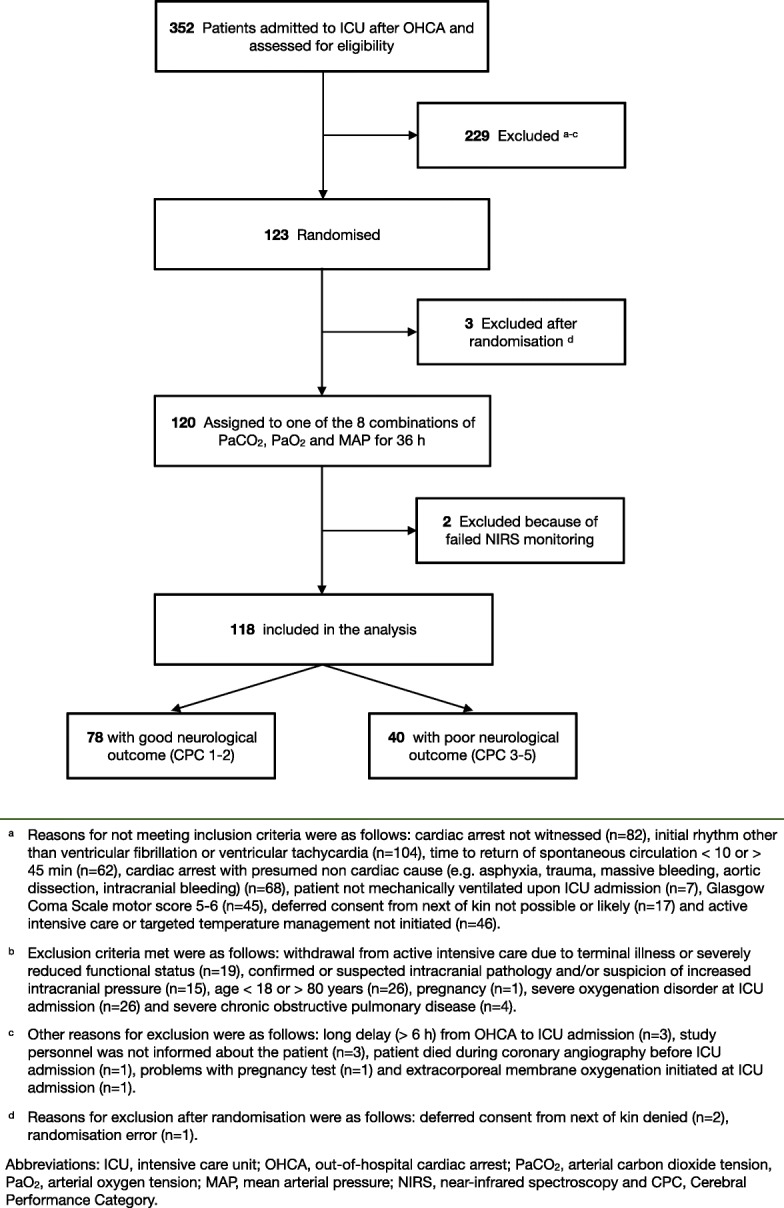
Screened, excluded and included patients in the study

The flowchart demonstrating patient enrolment is presented in Fig. 1. The patient recruitment began on 22 March 2016 and was completed by 3 November 2017. The 6-month-follow-up of the last patient was completed by 3 May 2018. The baseline characteristics and resuscitation-associated factors according to the 6-month neurological outcome are presented in Table 1.

**Fig. 2:**
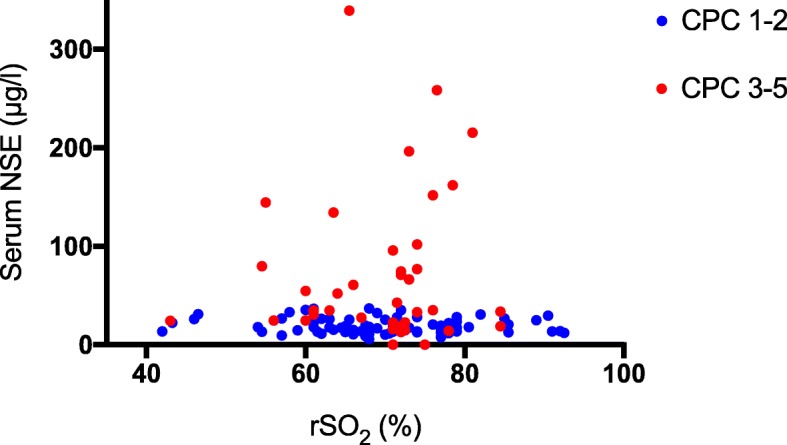
Scatter plots of serum neuron-specific enolase (NSE) concentration at 48 h after cardiac arrest vs. median regional cerebral oxygen saturation (rSO_2_) during the first 36 h in intensive care unit in patients with good (Cerebral Performance Category [CPC] 1–2) and poor (CPC 3–5) neurological outcome

The median (inter-quartile range (IQR)) NSE concentration at 48 h was 17.5 (13.4–25.0) μg/l and 35.2 (22.6–95.8) μg/l in patients with good and poor neurological outcome, respectively, *p* < 0.001. We found no statistically significant correlation between median rSO_2_ during the first 36 h in the ICU and serum NSE concentration at 48 h after CA, *r*_*s*_ = − 0.08, *p* = 0.392 (Fig. 2). In addition, there was no correlation between median rSO_2_ during the first 36 h in the ICU and serum NSE concentrations at 24 h or 72 h after CA (Additional file 1).

**Fig. 3:**
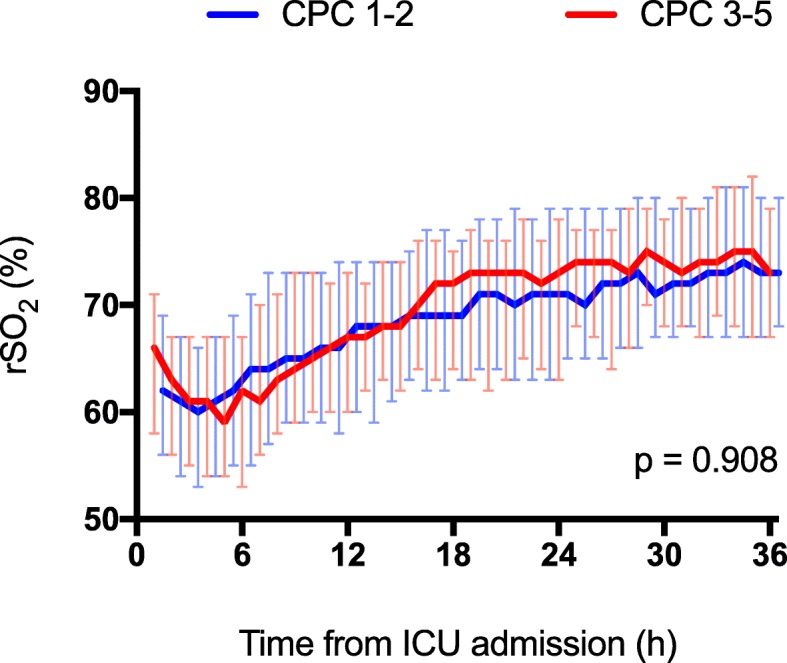
Median (inter-quartile range) regional cerebral oxygen saturation (rSO_2_) during the first 36 h of intensive care in patients with good (Cerebral Performance Category [CPC] 1–2) and poor (CPC 3–5) neurological outcome

The median (IQR) rSO_2_ during the first 36 h of intensive care was 70.0% (63.5–77.0%) in patients with good neurological outcome and 71.8% (63.3–74.0%) in patients with poor neurological outcome, *p* = 0.943. There was no significant association between rSO_2_ over time and neurologic outcome (Fig. 3). In the binary logistic regression model, the median rSO_2_ during the first 36 h in the ICU was not a statistically significant predictor of good outcome (adjusted odds ratio 0.99, 95% confidence interval 0.94–1.04, *p* = 0.635). Additionally, the worst 60-min median rSO_2_ did not associate with good neurological outcome (Table 2).

**Table 2:**
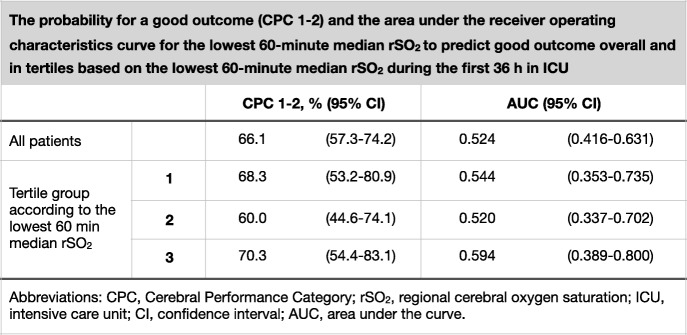
The probability for a good outcome (CPC 1–2) and the area under the receiver operating characteristic curve for the lowest 60-min median rSO_2_ to predict good outcome overall and in tertiles based on the lowest 60-min median rSO_2_ during the first 36 h in ICU

*CPC* cerebral performance category, *rSO2* regional cerebral oxygen saturation, *ICU* intensive care unit, *CI* confidence interval, *AUC* area under the curve

## Discussion

We prospectively monitored rSO_2_ with NIRS monitoring for 36 h after ICU admission in 118 patients resuscitated from OHCA with VF or VT as initial rhythm. This is, to our knowledge, the largest cohort of post-resuscitation patients with continuous NIRS monitoring during the early intensive care so far, and it is the only cohort including patients exclusively with shockable initial rhythms. We did not find any significant association between rSO_2_ and NSE concentrations at 48 h after CA, nor between rSO_2_ and neurological outcome at 6 months. Our findings question the role of routine NIRS monitoring in these patients.

Pursuing optimal brain oxygenation appears as an intuitively appealing strategy during post-resuscitation care [14, 15]. Despite its limitations, NIRS provides a non-invasive method to continuously monitor cerebral tissue oxygen saturation in a part of the frontal cortex and to detect clinically silent episodes of cerebral ischaemia [2]. Based on its already widespread use in different perioperative settings and the promising results of rSO_2_ as a predictor of ROSC during CPR, the expectations have been high that the information provided by NIRS could facilitate optimisation of the treatment of resuscitated patients and provide a feasible target for goal-directed therapy after CA.

The results of previous studies assessing the association between cerebral oxygenation during post-resuscitation care and outcome have been controversial [6–9]. We assessed the possible association between post-resuscitation rSO_2_ and long-term neurological outcome in a defined population of CA patients with shockable initial rhythm and a relatively good prognosis, with 66% of the patients achieving good neurological outcome. In previous studies that found an association between NIRS values and outcome after CA, the overall rate for good neurological recovery (CPC 1–2) has been 38–47% [7, 9]. It is possible that the larger proportion of patients with poor outcome in these cohorts than in our study population may explain the different results.

In the COMACARE trial, high-normal PaCO_2_ and moderate hyperoxia significantly increased rSO_2_ values during the first 36 h of intensive care [10]. However, there was no difference in serum NSE concentrations at 24, 48 or 72 h after CA or neurological outcome at 6 months between any of the intervention groups. This supports the view that achieving higher rSO_2_ does not necessarily improve chances for good recovery. In many surgical settings, NIRS is used as a perioperative monitor, and changes of 15–20% from the baseline values are considered meaningful [3]. By contrast, in CA patients, the normal baseline is unknown and the optimal or even minimal level of rSO_2_ for optimal recovery remains unidentified. In addition, it is possible that the extent of the neuronal damage during CA is the major determinant of outcome and all the secondary changes in cerebral blood flow (CBF) after ROSC have only a minor effect on the patient’s prognosis. One may also speculate that increasing oxygen delivery to the brain does not necessarily mean that the damaged neurons are able to utilise oxygen better [16].

RSO_2_ is thought to reflect CBF, and a recent study found a moderate correlation between rSO_2_ and cerebral perfusion pressure (CPP) [17]. However, the significance of optimising CPP and CBF to improve outcomes during post-resuscitation care remains undefined. In another study assessing CBF with serial duplex ultrasound measurements after CA, there was no association between CBF and outcome [18], which is in accordance with our finding of the lack of correlation between rSO_2_ and outcome. Interestingly, a correlation was found between the systemic blood pressure and CBF in patients with poor outcome, suggesting that impaired autoregulation of CBF during post-resuscitation care is related to poor prognosis. Impaired autoregulation during the first 24 h after CA has indeed been previously associated with poor outcome [19]. It is possible that rSO_2_ monitoring might be useful in the assessment of autoregulation, but more research is needed to determine its role.

The current study has several strengths. First, this was a large cohort of resuscitated patients with continuous NIRS monitoring. Second, we assessed the association of cerebral oxygenation with outcome in patients resuscitated from exclusively shockable initial rhythms. Third, we used continuous NIRS monitoring applied soon after ICU admission and continued for 48 h. Fourth, the physicians and ICU personnel responsible for the intensive care of the study patients were blinded to the NIRS monitor readings. Fifth, we treated all patients according to the current guidelines for post-resuscitation intensive care including targeted temperature management at either 33 °C or 36 °C. Finally, we studied patients in multiple centres and in two different countries.

Our study has some limitations. First, the design of this sub-study was conceived post hoc and was not included in the original COMACARE protocol [12]. Second, we did not assess CBF by transcranial Doppler ultrasound. As rSO_2_ is a surrogate indicator of CBF, transcranial Doppler could have provided additional information. Third, we used NIRS probes attached on the patients’ forehead which provides information about a small area of the frontal cerebral cortex, leaving other parts of the brain uncovered. Thus, we cannot exclude regional variation in rSO_2_ which could have caused bias to our results. Fourth, the fact that we studied a relatively selected population of CA patients with only shockable initial rhythms limits the generalisability of the results. Finally, although we studied patients in multiple centres, a majority were recruited in one hospital.

## Conclusions

We did not find any association between cerebral oximetry (rSO_2_) during the first 36 h of post-resuscitation intensive care and NSE serum concentrations at 48 h after OHCA or neurological outcome at 6 months.

## Additional file

Additional file 1: Association between rSO_2_ and the NSE concentrations at 24 h and 72 h. The scatterplots and the results of the Spearman rank order correlation analyses concerning the associations between rSO_2_ and the serum NSE concentrations at 24 h and 72 h after cardiac arrest. (PDF 171 kb)

## Abbreviations

APACHE: Acute Physiology and Chronic Health Evaluation
CA: Cardiac arrest
CBF: Cerebral blood flow
CPC: Cerebral performance category
CPP: Cerebral perfusion pressure
CPR: Cardiopulmonary resuscitation
ICU: Intensive care unit
IQR: Inter-quartile range
MAP: Mean arterial pressure
MWU: Mann-Whitney *U* test
NIRS: Near-infrared spectroscopy
NSE: Neuron-specific enolase
OHCA: Out-of-hospital cardiac arrest
PaCO_2_: Arterial carbon dioxide tension
PaO_2_: Arterial oxygen tension
ROSC: Return of spontaneous circulation
rSO_2_: Regional cerebral oxygen saturation
TTM: Targeted temperature management
VF: Ventricular fibrillation
VT: Ventricular tachycardia
