# Supplementary material for: Near-infrared spectroscopy after out-of-hospital cardiac arrest
Source: Crit Care. 2019 May 14;23:171. doi: 10.1186/s13054-019-2428-3 (PMC6518726; doi:10.1186/s13054-019-2428-3)
Supplement: Supplementary file 1 — Association between rSO2 and the NSE concentrations at 24 h and 72 h. The scatterplots and the results of the Spearman rank order correlation analyses concerning the associations between rSO2 and the serum NSE concentrations at 24 h and 72 h after cardiac arrest. (PDF 171 kb) [file 13054_2019_2428_MOESM1_ESM.pdf]

Near-infrared spectroscopy after out-of-hospital cardiac arrest

Authors

Pekka Jakkula, MD, Department of Anaesthesiology, Intensive Care and Pain Medicine, University of Helsinki and Helsinki University Hospital, Helsinki, Finland, pekka.jakkula@me.com, +358 400 552370 (corresponding author)

Johanna Hästbacka, MD, PhD, Adjunct Professor, Department of Anaesthesiology, Intensive Care and Pain Medicine, University of Helsinki and Helsinki University Hospital, Helsinki, Finland

Matti Reinikainen, MD, PhD, Adjunct Professor, Department of Anaesthesiology and Intensive Care, University of Eastern Finland and Kuopio University Hospital, Kuopio, Finland

Ville Pettilä, MD, PhD, Professor, Department of Anaesthesiology, Intensive Care and Pain Medicine, University of Helsinki and Helsinki University Hospital, Helsinki, Finland

Pekka Loisa, MD, PhD, Department of Intensive Care, Päijät-Häme Central Hospital, Lahti, Finland

Marjaana Tiainen, MD, PhD, Department of Neurology, University of Helsinki and Helsinki University Hospital, Helsinki, Finland

Erika Wilkman, MD, PhD, Adjunct Professor, Department of Anaesthesiology, Intensive Care and Pain Medicine, University of Helsinki and Helsinki University Hospital, Helsinki, Finland

Stepani Bendel, MD, PhD, Adjunct Professor, Department of Intensive Care, Kuopio University Hospital, Kuopio, Finland

Thomas Birkelund, MD, Aarhus University Hospital, Aarhus, Denmark

Anni Pulkkinen, MD, Department of Intensive Care, Central Finland Central Hospital, Jyväskylä, Finland

Minna Bäcklund, MD, PhD, Department of Anaesthesiology, Intensive Care and Pain Medicine, Helsinki University Hospital, Helsinki, Finland

Sirkku Heino, MD, Department of Anaesthesiology and Intensive Care, North Karelia Central Hospital, Joensuu, Finland

Sari Karlsson, MD, PhD, Adjunct Professor, Department of Intensive Care, Tampere University Hospital, Tampere, Finland

Hiski Kopponen, MD, Department of Anaesthesiology, Intensive Care and Pain Medicine, Helsinki University Hospital, Helsinki, Finland

Markus B Skrifvars, MD, PhD, Professor, Department of Emergency Medicine and Services, University of Helsinki and Helsinki University Hospital, Helsinki, Finland

Association between rSO_2 and the NSE concentrations at 24 h and 72 h

We found no statistically significant correlation between median rSO_2 during the first 36 h in the ICU and serum NSE concentration at 24 h after cardiac arrest, $r_s = 0.047$, $p = 0.610$ (Figure S1). Also, we found no statistically significant correlation between median rSO_2 during the first 36 h in the ICU and serum NSE concentration at 72 h after cardiac arrest, $r_s = -0.087$, $p = 0.367$ (Figure S2).

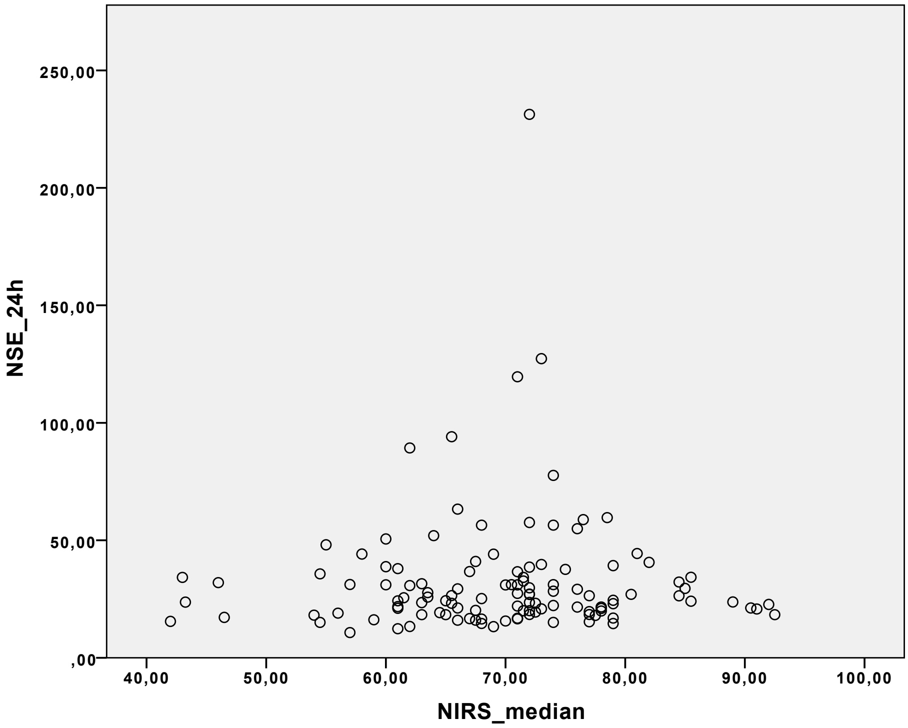

Figure S1 Scatter plots of serum neuron-specific enolase (NSE) concentration at 24 h after cardiac arrest vs. median regional cerebral oxygen saturation (rSO_2) during the first 36 h in intensive care unit

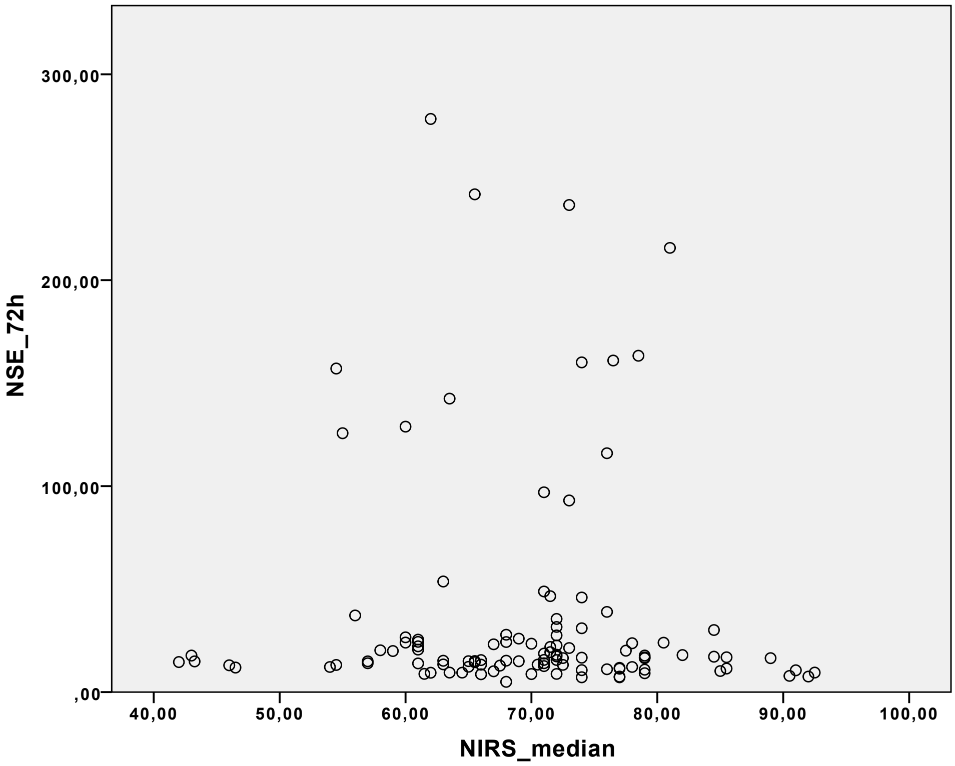

Figure S2 Scatter plots of serum neuron-specific enolase (NSE) concentration at 72 h after cardiac arrest vs. median regional cerebral oxygen saturation (rSO_2) during the first 36 h in intensive care unit
